# Prognostic Role of Vascular Endothelial Growth Factor and Correlation with Oxidative Stress Markers in Locally Advanced and Metastatic Ovarian Cancer Patients

**DOI:** 10.3390/diagnostics13010166

**Published:** 2023-01-03

**Authors:** Oana Gabriela Trifanescu, Laurentia Nicoleta Gales, Bogdan Cosmin Tanase, Serban Andrei Marinescu, Raluca Alexandra Trifanescu, Iuliana Maria Gruia, Mihai Andrei Paun, Laura Rebegea, Radu Mitrica, Luiza Serbanescu, Rodica Maricela Anghel

**Affiliations:** 1 Department of Oncology, University of Medicine and Pharmacy “Carol Davila”, 020021 Bucharest, Romania; 2 Radiotherapy II, “Prof. Dr. Al. Trestioreanu” Institute of Oncology Bucharest, 022328 Bucharest, Romania; 3 Medical Oncology II, “Prof. Dr. Al. Trestioreanu” Institute of Oncology Bucharest, 022328 Bucharest, Romania; 4 Thoracic Surgery, “Prof. Dr. Al. Trestioreanu” Institute of Oncology Bucharest, 022328 Bucharest, Romania; 5 Oncologic Surgery I, “Prof. Dr. Al. Trestioreanu” Institute of Oncology Bucharest, 022328 Bucharest, Romania; 6 “C. I. Parhon” Institute of Endocrinology, 011863 Bucharest, Romania; 7 Department of Endocrinology C. I. Parhon, University of Medicine and Pharmacy “Carol Davila”, 020021 Bucharest, Romania; 8 Medical Clinical Department, Faculty of Medicine and Pharmacy, “Dunărea de Jos” University, 800008 Galați, Romania

**Keywords:** vascular endothelial growth factor, malondialdehyde, ovarian adenocarcinoma

## Abstract

Background: Vascular endothelial growth factor (VEGF) plays an important role in tumor progression in ovarian cancer, but the complex mechanism and interaction with oxidative stress are not fully understood. Methods: A prospective study included 52 patients with ovarian adenocarcinoma stage IIIA-IV. Serum VEGF and reactive oxygen species (ROS) such as malondialdehyde and ceruloplasmin were measured. Results: VEGF levels were elevated (mean 1014.7 ± 165 pg/mL), especially in patients with macroscopic residual disease (1058 vs. 810 pg/mL, *p* = 0.0001). Median progression-free survival (PFS) and overall survival (OS) were 6 and 40 months in patients with a very high VEGF (over 1200 pg/mL), 11 and 48 months in patients with VEGF between 1000–1200 pg/mL, 18 and 84 months in patients with VEGF between 800–1000 pg/mL, and not reached in patients with normal VEGF. Increased VEGF values were associated with a 2.6-fold increased risk of disease progression (HR = 2.60, 95% CI 1.69–3.99), and a 1.4-fold increased risk of death (HR = 1.4, 95% CI 1.15–1.91, *p* = 0.002). Receiver operator characteristic (ROC) curves were used to validate VEGF as a prognostic factor and the area under the curve (AUC) was 0.814, *p* = 0.036 for PFS and 0.729, *p* = 0.043, for OS. There was a positive correlation between VEGF and malondialdehyde, Pearson coefficient of 0.35, *p* = 0.0001. Conclusions: VEGF and malondialdehyde are important prognostic markers in ovarian cancer, especially in macroscopic residual disease, and there is a positive correlation between angiogenesis and oxidative stress.

## 1. Introduction

Angiogenesis, the formation of new blood vessels from pre-existing ones, plays an important role in tumor growth, proliferation, and metastasis, and is one of the most important hallmarks of cancer [1,2,3].

Under normal conditions, angiogenesis is triggered by hypoxia and ischemia. Under the conditions of tumor angiogenesis, the process of new vessel formation is augmented by the preponderance of pro-angiogenic factors such as vascular endothelial growth factor (VEGF), placental growth factor (PlGF), and fibroblast growth factor (FGF), compared to anti-angiogenic factors such thrombospondin, angiostatin, endostatin, canstatin, and tumstatin [4].

VEGF-A is the most important pro-angiogenic factor and the one with the most clinical validity. Binding VEGF-A to VEGFR-1 and 2 which are overexpressed by endothelial cells leads to a cascade of activating signaling pathways. First, the dimerization of the receptor happens, followed by activation of the PLCγ–PKC–Raf kinase–MEK–MAPK pathway favoring DNA synthesis and cell growth, whereas activation of the phosphatidylinositol 3′–kinase (PI3K)–Akt pathway leads to increased endothelial-cell survival [5]. The effect is the upregulation of genes involved in the proliferation and migration of endothelial cells and promoting their survival and increasing vascular permeability. The angiogenic cascade enables a growing tumor to meet its increasing metabolic demands, but may also facilitate metastatic dissemination [6].

The hypoxic tumor micro-environment activates the angiogenic cascade, favoring the formation of new vessels in the tumor [4]. Hypoxia-inducible factors (HIF1α and 2α) mediate the transcription of VEGF messenger RNA and stimulate the down-regulation of several anti-angiogenic factors such as thrombospondin-1 and angiostatin [7].

Ovarian cancer cells express high levels of VEGF, mainly induced by hypoxia, suggesting that the same pathways are involved in tumor angiogenesis [8]. VEGFR-2 and platelet-derived growth factor receptors are involved in platinum resistance [9], and some studies found that post-operative elevated levels of VEGF correlate with poor outcomes [10].

A vast majority of oncogenes (such as Ras, Myc, C-Jun, and EGFR) and tumor suppressor genes (p53) are involved in the upregulation of vascular endothelial growth factor (VEGF), and in the downregulation of thrombospondin 1 (TSP-1) which inhibits angiogenesis [11].

ROS are produced in every cell as a result of oxygen consumption and cellular metabolism, due to partial reduction of oxygen [12,13]. Excessive production of ROS has been reported in different types of cancer, as well as in atherosclerosis, neurodegenerative diseases, and endometriosis, but the definitive role of ROS in ovarian cancer is still unknown [14,15,16].

It also has been shown that reactive oxygen species (ROS) have a direct effect on the stabilization of the hypoxia-inducible factor (HIF-1a), and antioxidants decrease the activation and accumulation of HIF-1a [17].

ROS act as signaling molecules, favoring tumor growth, metastasis, resistance to apoptosis, and angiogenesis [13]. On the other hand, ROS have an antitumorigenic effect, inducing oxidative stress-mediated tumor cell death. Oxidative stress is usually defined as an imbalance between the production of ROS and antioxidants and the ensuing pathophysiological consequences of increased, unspecified ROS [18,19].

This study aimed to measure VEGF and oxidative stress parameters, to determine if the presence of the tumor increases the production of VEGF, to validate VEGF as a prognostic factor, and to determine if there is a correlation between angiogenesis and ROS in patients with epithelial ovarian cancer.

## 2. Materials and Methods

### 2.1. Patients

We conducted a prospective study that included 52 patients diagnosed with stage III-IV epithelial ovarian carcinoma between January 2014 and January 2020.

The inclusion criteria were as follows: women at least 18 years of age, diagnosed with stage III-IV ovarian carcinoma who underwent multimodality treatment consisting of surgery with radical intent (total hysterectomy and bilateral oophorectomy) or biopsy, and chemotherapy with a platinum salt doublet (Paclitaxel 175 mg/m^2^ and Carboplatin AUC 5–6 every 3 weeks) [20,21]. Other criteria were ECOG performance status 0, 1, or 2, adequate blood count values (hemoglobin ≥ 9 g/dL, neutrophils ≥ 1500/mm^3^, platelets ≥ 100,000/mm^3^), and adequate values of biochemical parameters (creatinine clearance ≥ 50 mL/min, total bilirubin ≤ 3 mg/dL), willing to undergo chemotherapy. All patients signed the informed consent form approved by our institution for participating in this study.

Initial evaluation included complete blood count, evaluation of liver and renal function, CA125, CT or MRI of abdomen and pelvis.

Chemotherapy in the first line of treatment consisted of platinum-salt doublets. Bevacizumab and Olaparib were excluded, either due to them not being reimbursed according to local policy as part of the first-line treatment at the time of patients’ enrolment, or had contraindications to anti-VEGF treatment [22].

### 2.2. VEGF Assessment

Blood samples (5 mL) were obtained at the beginning of each chemotherapy cycle (for a total of 4 samples). Serum samples were obtained by venous puncture from peripheral venous blood and stored at −80 °C until analysis. The serum was isolated by centrifugation and the following determinations were performed: VEGF, lipid peroxidation, ceruloplasmin, thiols, and total antioxidants. VEGF levels were determined using a human VEGF enzyme-linked immunosorbent assay (ELISA; Sigma Aldrich kit from Merck, Wien, Austria) following the manufacturer’s instructions. All analyses were run in duplicate. The threshold level of normal serum VEGF was less than 800 pg/mL.

### 2.3. Malondialdehyde and Ceruloplasmin Assessment

Lipid peroxidation was assessed by measuring serum malondialdehyde (MDA) concentration. The Carbonneau spectrophotometric method was used, and this method is based on the production of a colored adduct (MDA-TBA2) with maximum absorption at 532 nm. Normal levels were between 0–4 μmol/100 mL serum [23,24].

Ceruloplasmin (CP) is an acute-phase copper-binding protein, mainly synthesized by the hepatocytes, which is a pro-inflammatory molecule elevated in infections, pregnancy, and trauma. As a multifunctional enzyme, ceruloplasmin exhibits amino-oxidase activity, superoxide dismutase, and ferro-oxidase activity. The oxidative activity of ceruloplasmin was determined using the Ravin spectrophotometric method based on the reaction between p-phenylenediamine and ceruloplasmin, and the color intensity developed at 540 nm was directly proportional to the concentration of ceruloplasmin. Normal values were between 80 and 120 I.U. [25].

### 2.4. Statistical Analysis

The statistical analysis was conducted using SPSS, version 23.0 for Windows, and all eligible patients were included. The oncologic outcome was calculated using the Kaplan–Meier method to determine PFS, defined as the time from cancer diagnosis to the disease progression on imaging or death from any cause, and median overall survival (OS) defined as the time from diagnosis to death of any cause. The univariate analysis using the log-rank test was used for analyzing the influence of different factors regarding the oncologic outcome, and a multivariate analysis was used according to the stepwise Cox proportional hazards model to identify independent prognostic factors and estimate their effect on the time to disease progression and overall survival. The *p* value was considered statistically significant if it was <0.05. Receiver operating characteristics (ROC) curves were used to measure the model’s efficacy, determine a prognostic cut-off value, and estimate the sensibility and specificity of the method. An area under the curve (AUC) closer to 1 is considered an efficient model and AUC values >0.6 validate the model.

The study was approved by the Local Ethical Committee of the Institute of Oncology (24644/2022). No specific informed consent form (ICF) was used because all patients signed the institutional ICF giving consent to medical procedures and full use of their medical records for research purposes. The study was conducted in harmonization with the World Medical Association (WMA) Helsinki Declaration of 1975, as revised in 2008.

## 3. Results

### 3.1. Oncologic Outcome

Fifty-two patients with confirmed ovarian adenocarcinoma were included in the study. The mean age at diagnostic was 52.4 ± 8.1 years (range 42–79). The stage distribution showed stage III with microscopic or macroscopic peritoneal metastasis in 67.3% and stage IV in 32.7% of the patients.

Median follow-up was 37 months, with a minimum of 9 months and a maximum of 121 months from the initial diagnosis. The median PFS for the entire group of patients was 21 months, and the median OS was 55 months.

The group was divided into two subgroups: subgroup A included 18 patients with stage III disease, where surgery was performed with curative intention and the postoperative evaluation no longer detected macroscopic residual disease; and subgroup B, which included 34 patients with stage III disease with macroscopic residual disease detected after surgery, and patients with stage IV or relapsed ovarian neoplasm.

### 3.2. VEGF as a Prognostic Factor

To better characterize the tumor in terms of antiangiogenic behavior and to determine if there is a connection between angiogenic signaling pathways represented by hypoxia and the oxygen free radicals, we determined the serum values of the vascular endothelial growth factor (VEGF) and oxidative stress parameters.

The serologic value of VEGF was determined before the first four cycles of chemotherapy. The mean value of VEGF measurement was 1014.7 ± 165 pg/mL and the median value was 979 pg/mL with a range between 704–1458 pg/mL. There was no statistical difference between the four determinations.

**Figure 1:**
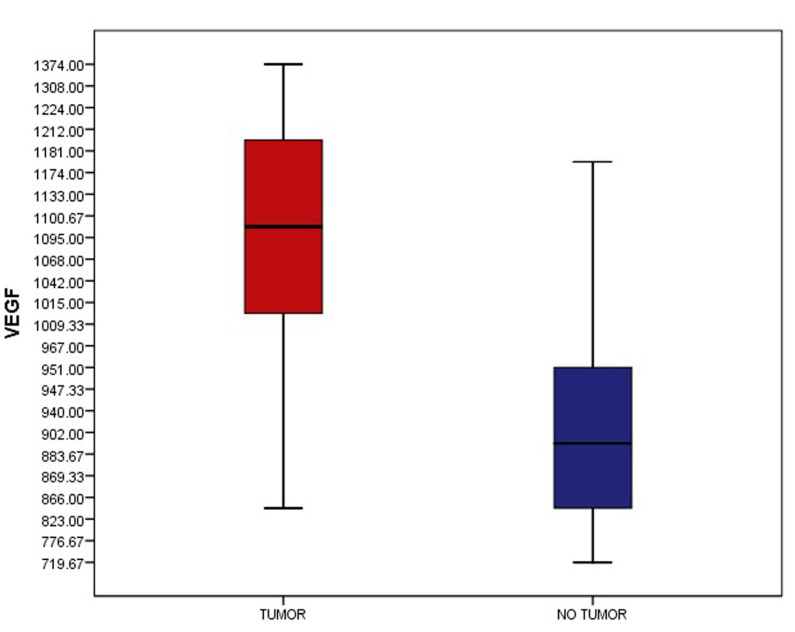
Serum VEGF levels in patients with macroscopic tumors vs. patients with no macroscopic tumors (*p* = 0.0001).

VEGF values were statistically different in patients with a residual macroscopic tumor compared with patients without any residual tumor. Thus, in the group of patients with residual tumors, the median value was 1070 pg/mL, and in the group of patients without tumors, their mean value was 897 pg/mL. There was a statistically significant difference between the two categories of values, *p* = 0.0001, as shown in Figure 1.

The present study aims to evaluate the prognostic role of VEGF in patients with ovarian adenocarcinoma. Patients were divided into four categories: patients with VEGF values below 800 pg/mL, patients with values between 800 pg/mL and 1000 pg/mL, patients with VEGF between 1000–1200 pg/mL, and patients with VEGF values over 1200 pg/mL.

**Figure 2:**
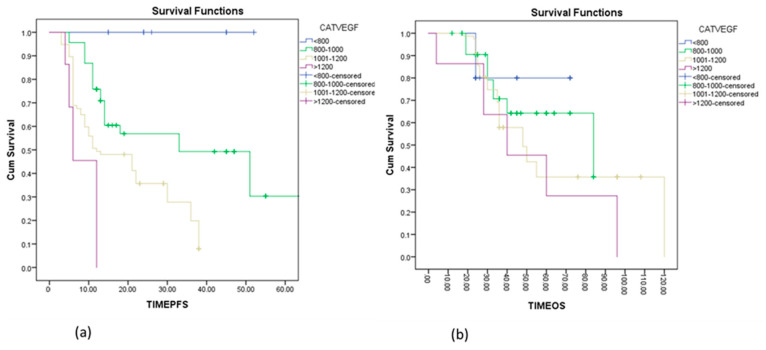
Kaplan–Meier curves estimating disease-free survival (**a**) and overall survival (**b**) according to VEGF categories (less than 800 pg/mL, 800–1000 pg/mL, 1001–1200 pg/mL, more than 1200 pg/mL) in patients with ovarian cancer.

The median interval until disease progression was 6 months in patients with a very high VEGF value (over 1200 pg/mL), compared to 11 months in patients with VEGF between 1000–1200 pg/mL, 32 months in patients with VEGF between 800–1000 pg/mL, and was not reached in patients with normal VEGF levels. Increased VEGF values were associated with a 2.7-fold increased risk of disease progression (HR = 2.79, 95% CI 2.193–3.565, *p* = 0.0001) (Figure 2a).

There was a trend to longer OS in patients with normal VEGF levels compared with patients with elevated VEGF; OS was not reached for patients with normal VEGF levels, 84 months for patients with VEGF between 800–1000 pg/mL, 48 months for patients with VEGF between 1000–1200 pg/mL, and 40 months for patients with VEGF more than 1200 pg/mL. Cox proportion hazard analysis showed a statistical increase in the risk of death in patients with elevated VEGF (HR = 1.4, 95% CI 1.15–1.91, *p* = 0.002) (Figure 2b).

**Figure 3:**
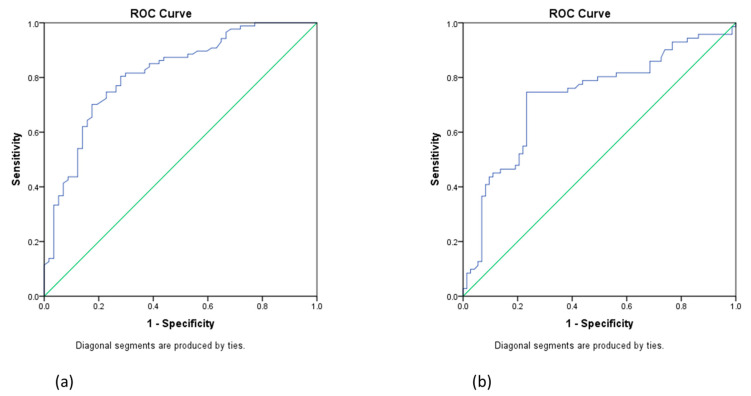
ROC curves of VEGF sensitivity and specificity for estimating the effect of VEGF on the time to disease progression (**a**) and overall survival (**b**).

To estimate the sensitivity and specificity of VEGF as a prognostic factor in ovarian cancer, we used ROC curve analysis. Thus, the area under the curve of VEGF for the estimation of the time until the disease progression was 0.814, *p* = 0.036, 95% CI 0.74–0.88. The cut-off value of VEGF to predict recurrence with 80% sensitivity and 30% specificity was 943 pg/mL (Figure 3a,b). Regarding OS, the area under the curve was 0.729, *p* = 0.043, 95% CI 0.64–0.81, and the cut-off value to predict survival with 80% sensibility and 50% specificity was 927 pg/mL.

### 3.3. ROS Measurement and Correlation with VEGF Levels

ROS were measured before each cycle of chemotherapy. The mean value of malondialdehyde in the whole group of 52 patients over four measurements was 7.85 μmol/100 mL serum ±2.92 (range 2.16 and 16.83 μmol/100 mL) compared with a normal value of less than 4 μmol/100 mL serum.

Ceruloplasmin levels were also consistently higher in the group of patients with ovarian cancer than the normal values, with mean levels of 176.81 ± 73.9 I.U. and a median of 159 I.U. compared with normal values, which are between 80 and 120 I.U.

Malondialdehyde and ceruloplasmin values were statistically significantly higher in patients in subgroup B with residual tumor as compared with patients in subgroup A without residual tumor (*p* = 0.0001 for malondialdehyde and *p* = 0.022 for ceruloplasmin, respectively).

**Figure 4:**
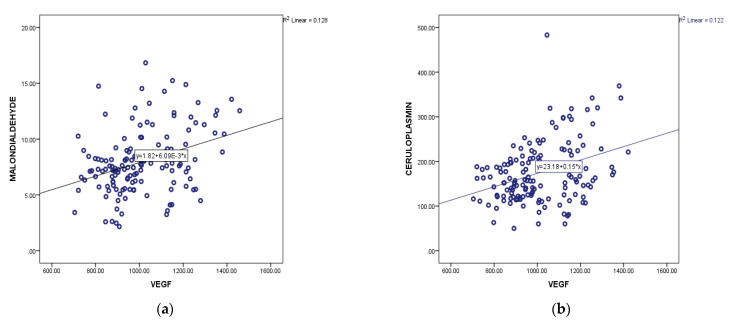
Positive correlation between VEGF and malondialdehyde (**a**) and VEGF and ceruloplasmin (**b**).

Starting from the experimental models that show a close interrelation between the reactive oxygen species signaling and angiogenesis, we studied whether there is a correlation between the values of the oxidative stress parameters and VEGF. There was a linear increase in VEGF values in positive correlation with lipid peroxidation markers (malondialdehyde) so that the Pearson correlation coefficient was 0.35 with statistical significance, *p* = 0.0001 (Figure 4a).

There is also a positive correlation between the value of ceruloplasmin and VEGF (Pearson coefficient 0.29 and *p* = 0.0001) (Figure 4b).

In our series of patients, there was a positive correlation between the malondialdehyde and ceruloplasmin values (Pearson correlation coefficient 0.38, *p* = 0.0001).

## 4. Discussion

Although there are numerous studies regarding VEGF and ROS separately in patients with various neoplasms, this study aims to evaluate angiogenesis and oxidative stress, the relationship with clinical and paraclinical prognostic factors, and the relationship between angiogenesis and oxidative stress in ovarian cancer patients.

The serum VEGF values obtained in our group of patients were much higher than the normal values. Elevated VEGF values were also associated with a shorter interval in disease progression and overall survival.

Chen et al. aimed to evaluate the relationship between VEGF and clinical risk factors in patients with ovarian cancer [26]. In 56 patients with stage I-IV ovarian adenocarcinoma, serum VEGF was measured preoperatively. The mean serum VEGF was 485.7 pg/mL. Elevated VEGF values were statistically significantly correlated with disease-free progression (DFS) and OS.

One study tested the influence of VEGF expression and blood micro-vessel density in the prognosis of 64 patients with ovarian carcinoma and found that VEGF overexpression was associated with poorer prognosis and statistically significantly lower survival than those without or with low VEGF expression [8].

A systematic review including studies that have measured serum VEGF in ovarian cancer has shown that there is a statistically significant association between VEGF level and FIGO stage, degree of tumor differentiation, size of the tumor, residual disease after surgery, lymph node invasion and ascites, but also showed that there are numerous conflicting studies regarding the prognostic value of VEGF [27]. Tempfer [19] and Li [20] showed that elevated serum VEGF values correlated with less differentiated tumors (G2 and G3 vs. G1) [28,29]. Li showed that serum VEGF values were higher in patients with residual disease after surgery.

The studies conducted by Chen, Li, and Tempfer showed that elevated serum VEGF values correlated with lower OS [26,28,29]. These studies are in opposition with Gadducci’s data which found no statistically significant difference in survival in the group of those with elevated serum VEGF vs. normal limits VEGF, and Mahner’s study did not find a statistically significant difference in the interval until disease progression [10,30].

Li’s subgroup analysis showed that there are no statistically significant differences in the survival of patients with stages I and II regarding serum VEGF values, but the OS for stages III and IV with elevated serum VEGF values was lower [20]. A more recent analysis showed that the mean value of serum VEGF-A was inversely proportional to the FIGO stage [31].

In a meta-analysis published in the Gynecologic Oncology Journal, which included 16 studies and a total of 1111 patients (385 analysis of serum and 638 analysis of ovarian tumor specimens), elevated VEGF values were associated with a shorter disease progression interval (HR = 2.46) and lower survival (HR = 1.7) [32].

The most recent meta-analysis included 1145 patients and showed that increased tissue VEGF expression is associated with decreased survival and lower PFS (for OS, HR = 2.24, 95% CI 1.36–3.70, *p* = 0.002, and for PFS HR = 1.60, 95% CI 1.11–2.31, *p* = 0.01) [33].

A study that measured VEGF-A values for 128 patients with ovarian cancer found that the values for stage I and II were statistically significantly lower than for stage III/IV (*p* = 0.0036), and that patients with increased VEGFR-2 values have a better prognosis than those with low values (HR = 2.01) [34]. Another study found that elevated levels of VEGF promote tumor growth and facilitate the spread of neoplastic cells in the abdominal cavity diminishing endothelial cell adhesion (VE-cadherin and Claudin 5), thus increasing vascular permeability and ascites production [35].

These discordant results obtained by different studies may have some explanations. First of all, a lot of studies were retrospective and included a limited number of patients with different characteristics. It has been shown that in patients with stages I and II or without residual disease after surgery, VEGF does not always have a prognostic value [29,34]. However, in patients with advanced stage and residual disease, serum VEGF correlates with the extent of the disease. Another factor of discordancy is the sample evaluated. Some studies evaluate serum VEGF, while others determine VEGF expression using RT-PCR or immunohistochemistry from the tissue sample. Meta-analysis concludes that for advanced-stage, serum VEGF is a less heterogenous determination, and is more predictive compared to immunohistochemical determination of VEGF [32]. The lack of a clear cut-off to determine low/elevated levels of VEGF and the difference in reporting immunohistochemical staining for VEGF may be another explanation.

VEGF can simultaneously promote angiogenesis and mediate immunosuppression by recruiting immunosuppressive cells, inhibiting the dendritic cell maturation, induction the inhibitory cell expression (PD-L1), and activation of T-reg [36].

In the neoplastic cell, the imbalance between the production of ROS and the elimination of free radicals leads to a state of oxidative stress and the destruction of essential components of the cell [37]. Several studies show that the oxidative stress resulting from this imbalance has a causative role, but it is also a consequence of carcinogenesis, interfering with all phases of this process, such as initiation, promotion, progression, invasion, and metastasis [11,38].

ROS activates pro-tumorigenic signaling, by increasing the rate of cell proliferation, increasing the resistance and survival of neoplastic cells, increasing hypoxia, and promoting gene instability and damage to genetic material [39]. As a compensatory mechanism, tumor cells express high levels of antioxidants to detoxify this excess amount of ROS and restore redox balance, but there is still a pro-tumorigenic status that promotes hypoxia, resistance to chemotherapy and apoptosis, and ovarian cancer patients have elevated levels of oxidative stress and low levels of antioxidants [40].

Data about ROS levels (malondialdehyde, carbonyl groups, and total antioxidants) in patients with ovarian carcinoma is scarce. A study that included patients with different ovarian masses (35 in total, 11 ovarian stages III and IV serous carcinoma) concluded that ROS concentration was elevated (96% higher) in the group of patients with ovarian carcinoma compared to the control arm. Excessive ROS production and elevated malondialdehyde levels exceed the capacity of the antioxidant systems to eliminate them, and might be a direct causal factor for the initiation and progression of ovarian serum carcinoma [41].

Patients with residual disease after surgery and inoperable stage IV have a poor prognosis [42]. In our series of patients, there was an increased level of VEGF and ROS, especially in patients with the macroscopic residual disease [43].

As a homeostatic mechanism against ROS production, an antioxidant enzymatic system represented by superoxide dismutase, catalase, and glutathione S-transferase must be activated. Several studies reported that in patients with high-grade ovarian serous carcinoma this response is dysregulated and low levels of glutathione peroxidase 3 were recorded in patients with metastatic or relapsed ovarian cancer [44,45].

Under hypoxia, the ROS levels increase mainly due to the component produced at the mitochondrial level [46]. Studies show that ROS originating in the mitochondria are necessary and sufficient to activate and stabilize HIF-1α [47]. Under hypoxic conditions, ROS activates signaling pathways upstream of HIF such as ERK and p38, with MAP-Kinase increasing HIF transcriptional activity. The incomplete mechanism elucidated so far assumes that p38α is activated by ROS and contributes to HIF-1α activation by inhibiting hydroxylation by prolyl hydroxylases and asparaginase [19].

HIF regulation is also achieved by modulating the phosphatidyl-inositol-3-kinase (PI3K)–Akt pathway. The PI3K–AKT pathway has several downstream effectors such as FOXO4, glycogen synthetase kinase 3 (GSK3), a human oncoprotein HDM2 (an important regulator of the tumor suppressor gene p53), and tuberin (TSC2) an important component of the mTOR pathway, all influencing HIF-1 activity [19,48,49,50].

A study showed that there is a direct correlation between VEGF and ROS derived from NADPH oxidase NOX, validating the role of ROS as pro-angiogenic signaling molecules [51]. In our study, there was a positive correlation between malondialdehyde values and VEGF values, showing a direct relation between angiogenesis and oxidative stress in ovarian cancer patients.

## 5. Conclusions

In patients with confirmed ovarian cancer, VEGF, malondialdehyde, and ceruloplasmin had prognostic value, both in terms of progression-free survival and overall survival, especially in patients with the residual macroscopic disease. There was a strong positive correlation between VEGF and malondialdehyde and ceruloplasmin. All these biomarkers may help identify patients with poor prognostic who need treatment escalation.

## Data Availability

Not applicable.
